# Association between apolipoprotein B/A1 ratio and quantities of tissue prolapse on optical coherence tomography examination in patients with atherosclerotic cardiovascular disease

**DOI:** 10.1007/s10554-023-03023-5

**Published:** 2024-01-10

**Authors:** Yao Du, Binbin Zhu, Yahui Liu, Zhou Du, Jianhong Zhang, Wei Yang, Huiping Li, Chuanyu Gao

**Affiliations:** Department of Cardiology, Zhengzhou University Central China Fuwai Hospital, No. 1 Fuwai Road, Zhengzhou, 451464 Henan China

**Keywords:** Apolipoprotein B/A1 ratio, Tissue prolapse, Optical coherence tomography

## Abstract

Atherosclerotic cardiovascular disease (ASCVD) continues to be a major health concern globally. Apolipoprotein (Apo) B/A1 ratio is a reliable predictor of ASCVD and an important factor in assessing the risk of myocardial infarction. Tissue prolapse (TP) is defined as the tissue extrusion into the lumen through the stent struts after implantation, which is a significant factor for poor short-term outcomes such as acute and subacute thrombosis, severe myocardial necrosis, and vulnerable plaque. Therefore, the aim of this study was to investigate the relationship between Apo B/A1, plaque vulnerability, and tissue prolapse on optical coherence tomography (OCT). This study enrolled 199 patients with atherosclerotic cardiovascular disease (ASCVD) who underwent percutaneous coronary intervention (PCI). Both pre- and post-procedural optical coherence tomography (OCT) examinations were conducted to assess TP volume and plaque morphology. Logistic regression analyses were performed to identify potential risk factors for tissue prolapse volume. Receiver operator characteristic (ROC) curve analysis was carried out to evaluate the value of the Apo B/A1 ratio for tissue prolapse volume. The high Apo B/A1 ratio group showed a larger TP volume (P = 0.001) and a higher percentage of plaque rupture and erosion in comparison to the low Apo B/A1 ratio group (P = 0.022 and P = 0.008). The high Apo B/A1 ratio group and the high TP volume group also had a higher proportion of thin-cap fibroatheroma (TCFA) (P = 0.046, P = 0.021). Multivariate logistic regression analysis revealed that both Apo B/A1 ratio (odds ratio [OR]: 1.041, 95% confidence interval [CI] 1.007–1.076; P = 0.019) and TCFA (OR: 3.199, 95%CI 1.133–9.031; 0.028) were significantly related to high TP volume. Furthermore, the area under the curve (AUC) for predictive value of TP volume was 0.635 for Apo B/A1 (95% CI 0.554–0.717, P = 0.002) compared to 0.615 for low density lipoprotein cholesterol (LDL-C) (95% CI 0.533–0.697, P = 0.008). The Apo B/A1 ratio is an independent predictor of TP volume on OCT and is related to plaque vulnerability.

## Background

While statin therapy is effective in achieving treatment objectives, the residual cardiovascular risk remains significantly high at approximately 70% [1, 2]. As a result, researchers have sought to identify more advanced biomarkers to help explain this residual risk. Apolipoproteins B and A1 are the main surface proteins on LDL and HDL particles, respectively. Thus, the apo B/A1 ratio may reflect the cholesterol balance between atherogenic and anti-atherogenic lipoprotein particles. Previous research has shown apo B/A1 ratio can predict CVD and is strongly associated with the risk of myocardial infarction [3, 4]. Additionally, the apo B/A1 ratio is associated with vulnerable plaque, including plaque rupture, erosion, and thrombus [5].

TP refers to refers to tissue extrusion into the lumen through stent struts after implantation [6]. Several intravascular ultrasound (IVUS) studies have demonstrated an association between tissue prolapse and poor short-term prognosis, including acute and subacute thrombosis and more myocardial necrosis [7–9]. OCT has a higher resolution than IVUS and can provide clearer and more reliable information on TP [10, 11]. However, the relationship between apo B/A1 ratio and TP has not been fully investigated. In this study, researchers assessed the TP volume detected by OCT and investigated the association between the apo B/A1 ratio and TP volume.

## Methods

### Patient population

This study is a single-center, cross-sectional analysis. Researchers retrospectively enrolled patients diagnosed with ASCVD and undergoing PCI with pre- and post-procedural OCT examination from December 2019 to November 2022 at Central China Fuwai Cardiovascular Hospital, and these patients were required to have implanted at least one stent in the target vessel. Exclusion criteria included end-stage renal disease, serious liver dysfunction, hematological disease, malignant tumor disease, allergy to statins, and poor OCT imaging quality. In addition, some AMI patients who potentially had a greater amount of atherosclerotic plaques and intracoronary thrombus were excluded in this study. Patients with severe coronary stenosis which resulted in failure of OCT catheter to pass or poor OCT imaging quality before balloon dilatation were also excluded. The study protocol was approved by the Human Research Committee of Fuwai Central China Cardiovascular Hospital.

### Clinical and laboratory data collection

Detailed demographic information, medical history, biochemical examination, drug usage, and angiographic data were obtained from the medical records. Venous blood samples were taken for serum lipid analysis when patients were hospitalized. Relevant lipoprotein markers such as LDL-C, high-density lipoprotein cholesterol (HDL-C), total cholesterol (TC), and triglycerides (TG) were measured by electro-chemiluminescence immunoassay. Other laboratory parameters were measured using standard methods upon admission.

### Angiographic procedure

All patients were treated with aspirin (300 mg loading dose, followed by 100 mg/day) and clopidogrel (300 mg loading dose, followed by 75 mg/day) or ticagrelor (180 mg loading dose, followed by 180 mg/day). Coronary angiography was performed via the transradial or transfemoral approach with a 6F or 7F sheath. Before PCI, intravascular infusion of 100–120 IU/kg unfractionated heparin was given. The culprit vessel was determined by a combination of coronary angiography, left ventricular wall motion abnormalities, electrocardiogram, and scintigraphic evidence of myocardial ischemia. Experienced interventionists selected the PCI strategy, and all patients underwent stent implantation with less than 25% residual stenosis on quantitative coronary angiography analysis.

### OCT image acquisition and analysis

The OCT system used in this study was frequency-domain OCT (C7 ILMIEN system; St. Jude Medical, St. Paul, MN, USA). To avoid the effect of coronary spasms, 0.2 mg nitroglycerin was injected into coronary arteries. After the accomplishment of coronary angiography, OCT examination was performed via an imaging catheter in a virtually blood-free environment. The catheter was advanced distal to the lesion and then pulled back by the machine automatically at a steady rate. OCT images were analyzed at every frame using an offline review workstation.

**Fig. 1:**
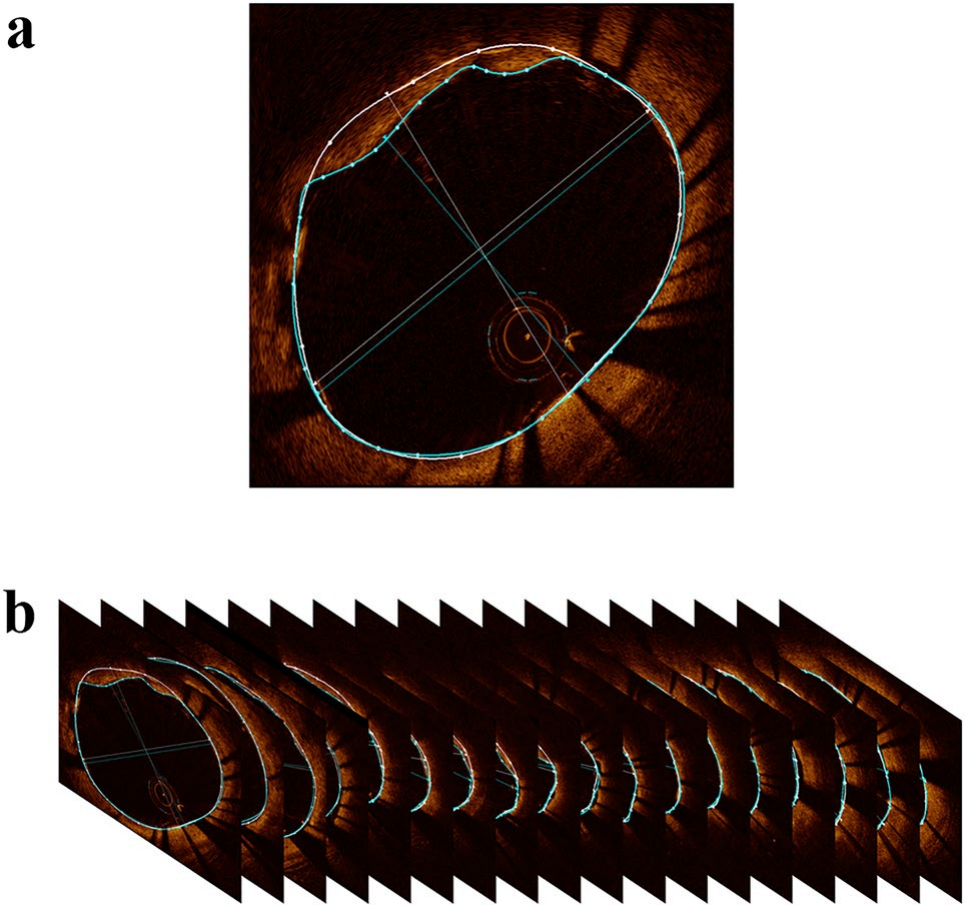
Measurement of TP volume on OCT. **A** TP area was calculated by subtracting the lumen area (blue line) from the stent area (white line); **B** TP volume was the sum of TP area measured in the whole stented segment at 1-mm intervals in the stented segment

**Fig. 2:**
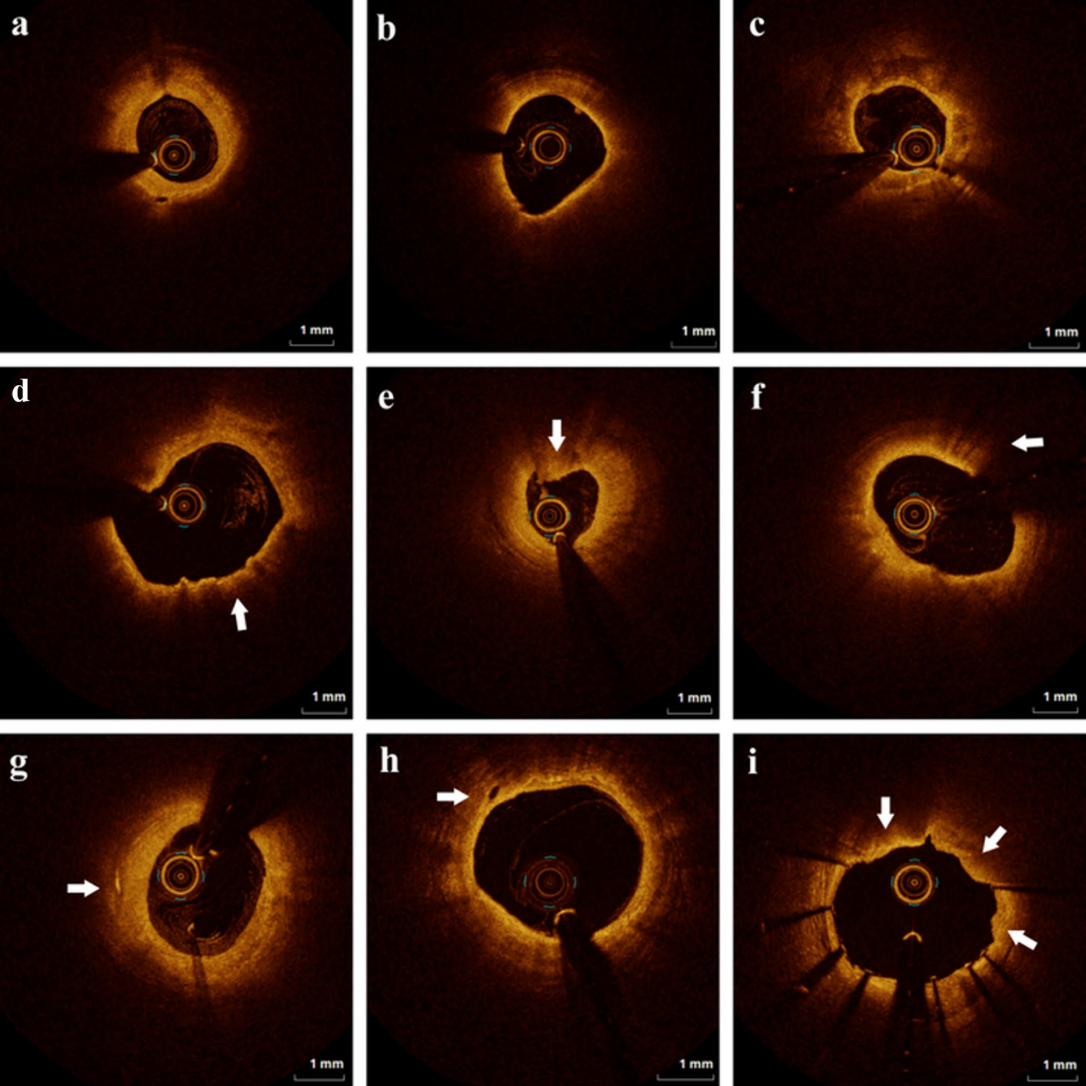
Representative optical coherence tomography images of various culprit vessel morphologies. **A** The fibrous plaque was defined as a plaque with homogeneous and highly backscattering regions. **B** Lipid-rich plaque was defined as a plaque with lipid arc > 180°. **C** Calcified plaque was defined as a plaque with calcification arc > 90° at the largest part. **D** Thin-cap fibroatheroma (TCFA) was defined as a plaque with lipid rich arc > 180° and the thinnest fibrous cap < 65 μm. **E** Plaque erosion was defined as the composed of evidence of thrombus, an irregular luminal surface, and no evidence of cap rupture evaluated in multiple adjacent frames. **F** Macrophage was defined as signal-rich punctuate region with heterogeneous backward shadows. **G** Cholesterol crystal was defined as thin, linear regions of high intensity, usually associated with a fibrous cap or necrotic core. **H** Microvessels were defined as black holes within a plaque with the presence on at least three consecutive frames. **I** Tissue prolapse was defined as the tissue extrusion into the lumen through the stent struts after implantation

Plaque morphology was characterized both qualitatively and quantitatively based mainly on previous criteria for OCT plaque characterization [6]. The presence of TP was evaluated immediately after PCI. TP area was assessed at 1-mm intervals throughout the stented segments, and plaque morphology features at the most protruding sites were evaluated, including plaque types, macrophage, cholesterol crystal, microvessels and TCFA. Fibrous plaque was defined as a plaque with homogeneous and highly backscattering regions. Lipid-rich plaque was defined as a plaque with a lipid arc greater than 180°. Calcified plaque was defined as a plaque with a calcification arc greater than 90° at the largest part, and calcium arc was measured by using the offline review workstation at 1-mm intervals. The lipid arc and the overlying fibrous cap thickness at the thinnest part through the whole lesion were measured at 1-mm intervals before PCI. The fibrous cap thickness of a plaque was the average value of three measurements. TCFA was defined as a plaque with a lipid rich arc greater than 180° and the thinnest fibrous cap less than 65 μm. Plaque rupture was defined as the discontinuous fibrous cap with obvious cavity formation, while plaque erosion was defined as the composed of evidence of thrombus, an irregular luminal surface, and no evidence of cap rupture evaluated in multiple adjacent frames. Macrophage was defined as a signal-rich punctuate region with heterogeneous backward shadows. Cholesterol crystal was defined as thin, linear regions of high intensity, usually associated with a fibrous cap or necrotic core. Microvessels were defined as black holes within a plaque with the presence of at least three consecutive frames. Thrombus by OCT appears as a mass attached to luminal surface or floating within the lumen. Red thrombus is highly backscattering and has a high attenuation (resembles blood), and white thrombus is less backscattering, is homogeneous, and has low attenuation. Tissue prolapse was defined as the tissue extrusion into the lumen through the stent struts after implantation. TP area was calculated by subtracting the lumen area from the stent area and TP volume was the sum of TP area measured in the whole stented segment at 1-mm intervals in the stented segment, as shown in Fig. 1. Representative OCT images of the culprit vessel were shown in Fig. 2. All OCT images were analyzed by two independent and experienced investigators who were blinded to the angiographic results and clinical data. A consensus diagnosis was obtained with the help of the third investigator if there was any disagreement between the two observers.

### Statistical analysis

IBM SPSS Statistics 26.0 software (SPSS Inc, Chicago, IL, USA) was used for all analyses. Categorical data were expressed as absolute frequencies and percentages (%) and compared using the chi-square test or Fisher’s exact test. Continuous data were expressed as mean ± standard deviation (SD) and compared using Student’s *t*-test, Mann–Whitney test, one-way analysis of variance, or Kruskal–Wallis test between two groups. Correlations between two variables were determined using the Pearson test or Spearman’s rank test as appropriate. The patients were also divided into two groups according to the median value of TP volume to explore the differences of OCT findings [12]. Logistic regression analyses were performed to assess risk factors for tissue prolapse volume. ROC curve analysis were performed to assess the value of the Apo B/A1 ratio for tissue prolapse volume. P value < 0.05 was considered statistically significant.

## Results

### Patient characteristics

**Fig. 3:**
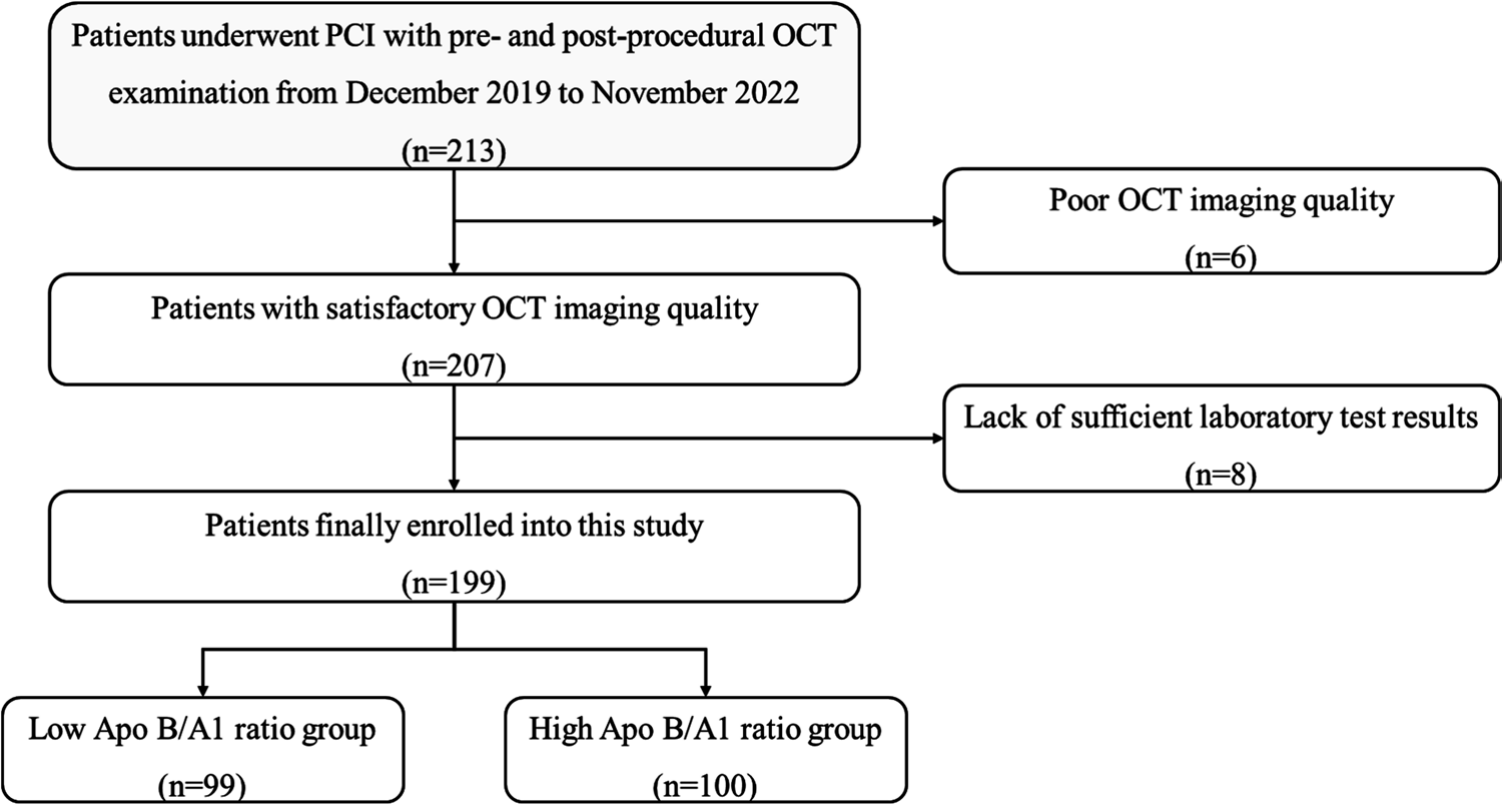
Flow chart of this study. *PCI* percutaneous coronary intervention, *OCT* optical coherence tomography, *Apo* apolipoprotein

213 patients who underwent PCI with pre- and post-procedural OCT examination from December 2019 to November 2022 in Central China Fuwai Cardiovascular hospital were enrolled in this study. 6 patients with poor OCT imaging quality and 8 patients who had insufficient laboratory test results were excluded. The patients were divided into two groups: the low Apo B/A1 ratio group (n = 99) and the high Apo B/A1 ratio group (n = 100), as shown in Fig. 3.

Baseline clinical characteristics between the two groups were displayed in Table 1. No significant differences were noted in terms of gender, age, and smoking history. However, a higher number of heart failure patients were observed in the high Apo B/A1 ratio group (P = 0.003). The left ventricular ejection fraction was significantly lower in the high Apo B/A1 ratio group (P = 0.039) and post-PCI CK-MB was significantly higher in the high Apo B/A1 ratio group (P = 0.020). Moreover, renal function biomarkers including creatine and uric acid were higher in the high Apo B/A1 ratio group (P < 0.05). There were no significant differences in statin medication taken at admission between the two groups (P = 0.398).

**Table 1:** Baseline characteristic of patients in the low and high Apo B/A1 ratio groups

Characteristics	All (n = 199)	Low ratio (n = 99)	High ratio (n = 100)	P value
Male, n(%)	142(71.4%)	67(67.7%)	75(75%)	0.253
Age, years, mean ± SD	60.08 ± 10.52	60.32 ± 10.22	59.84 ± 10.87	0.782
ACS	86(43.2%)	38(38.4%)	48(48.0%)	0.171
Medical history, n(%)				
Atrial fibrillation	1(0.5%)	1(1, 0%)	0(0%)	0.497
Hypertension	111(55.8%)	53(53.5%)	58(58.0%)	0.526
Diabetes mellitus	50(25.1%)	22(22.2%)	28(28.0%)	0.347
Heart failure	15(7.5%)	2(2.0%)	13(13.0%)	0.003
Stoke	19(9.5%)	9(9.1%)	10(10.0%)	0.827
Prior MI	24(12.1%)	14(14.1%)	10(10.0%)	0.370
Prior PCI	31(15.6%)	18(18.2%)	13(13.0%)	0.314
Dyslipidemia	51(25.6%)	26(26.3%)	25(25.0%)	0.838
Family history	44(22.1%)	20(20.2%)	24(24.0%)	0.519
Alcohol drinking	86(43.2%)	42(42.4%)	44(44.0%)	0.822
Smoking	90(45.2%)	42(42.4%)	48(48.0%)	0.429
Laboratory results, mean ± SD				
WBC, × 10^9^	6.78 ± 2.17	6.44 ± 1.76	7.12 ± 2.48	0.056
Hb, g/L	136.95 ± 16.51	134.49 ± 16.78	139.44 ± 15.95	0.065
HbA1c, %	6.14 ± 1.10	5.87 ± 0.83	6.41 ± 1.27	0.005
CK-MB, U/L	18.81 ± 18.08	16.64 ± 13.56	20.92 ± 21.55	0.251
Post-PCI CK-MB, U/L	18.50 ± 16.71	15.38 ± 12.16	21.66 ± 19.90	0.020
CRP, mg/L	2.40 ± 2.41	2.37 ± 2.31	2.44 ± 2.43	0.871
Creatine, μmol/L	71.19 ± 20.70	67.60 ± 16.93	74.78 ± 23.45	0.035
Uric acid, μmol/L	312.36 ± 86.31	296.87 ± 68.67	327.85 ± 98.98	0.029
TC, mmol/L	3.54 ± 0.79	3.18 ± 0.65	3.89 ± 0.76	< 0.001
TG, mmol/L	1.64 ± 1.02	1.42 ± 1.00	1.87 ± 0.99	0.007
HDL-C, mmol/L	1.00 ± 0.23	1.08 ± 0.24	0.91 ± 0.17	< 0.001
LDL-C, mmol/L	2.01 ± 0.64	1.65 ± 0.50	2.37 ± 0.57	< 0.001
Apo A1, g/L	1.11 ± 0.24	1.19 ± 0.26	1.02 ± 0.17	< 0.001
Apo B, g/L	0.70 ± 0.21	0.56 ± 0.13	0.84 ± 0.17	< 0.001
Apo B/A1, %	0.65 ± 0.23	0.48 ± 0.10	0.83 ± 0.18	< 0.001
LVEF, %	53.35 ± 19.45	56.61 ± 16.96	50.09 ± 21.27	0.039
Culprit vessels, n(%)				
LAD	156(78.4%)	79(79.8%)	77(77.0%)	
LCX	16(8.0%)	11(11.1%)	5(5.0%)	
RCA	37(18.6%)	18(18.2%)	19(19.0%)	
Lesion site, n(%)				
Proximal	128(64.3%)	68(68.7%)	60(60.0%)	
Middle	62(31.2%)	31(31.3%)	31(31.0%)	
Distal	19(9.5%)	9(9.1%)	10(10.0%)	
Stent, n(%)				
1	134(67.3%)	63(63.6%)	71(71.0%)	
2	51(25.6%)	29(29.3%)	22(22.0%)	
3	11(5.5%)	7(7.1%)	4(4.0%)	
4	3(1.5%)	1(1.0%)	2(2.0%)	
Statins, n(%)	(%)	68(68.7%)	63(63.0%)	0.398

*ACS* acute coronary syndrome, *SD* standard deviation, *MI* myocardial infarction, *PCI* percutaneous coronary intervention, *WBC* white blood cell, *Hb* hemoglobin, *HbA1c* glycosylated hemoglobin, *CK-MB* creatine kinase-MB, *CRP* C-reaction protein, *TC* total cholesterol, *TG* total triglycerides, *HDL-C* high-density lipoprotein cholesterol, *LDL-C* low-density lipoprotein cholesterol, *Apo* apolipoprotein, *LVEF* left ventricular ejection fraction, *LAD* left anterior descending artery, *LCX* left circumflex artery, *RCA* right coronary artery

### OCT findings of culprit vessels

OCT findings between the low and high Apo B/A1 ratio groups were shown in Table 2. The percentage of plaque rupture and plaque erosion was higher in the high Apo B/A1 ratio group (P = 0.022 and P = 0.008). Moreover, the proportion of TCFA was also higher in the high Apo B/A1 ratio group (P = 0.046). No significant differences were found among the fibrous plaque type, lipid-rich plaque type, and calcification plaque type. In terms of tissue prolapse, TP volume was larger in the high Apo B/A1 ratio group (P = 0.001).

**Table 2:** Optical coherence tomography characteristic of patients in the low and high Apo B/A1 ratio groups

Characteristics	All (n = 199)	Low ratio (n = 99)	High ratio (n = 100)	P value
Plaque morphology, n(%)				
Plaque rupture	32(16.1%)	10(10.1%)	22(22.0%)	0.022
Plaque erosion	32(16.1%)	9(9.1%)	23(23.0%)	0.008
Plaque type, n(%)				
TCFA	57(28.6%)	22(22.2%)	35(35.0%)	0.046
Fibrous plaque	54(27.1%)	24(24.2%)	30(30.0%)	0.361
FCT of fibrous plaque, μm, mean ± SD	154.50 ± 102.28	152.20 ± 101.79	156.80 ± 103.98	0.846
Lipid-rich plaque	97(48.7%)	47(47.5%)	50(50.0%)	0.722
FCT of lipid-rich plaque, μm	94.90 ± 62.56	85.60 ± 32.86	102.60 ± 79.71	0.404
Lipid arc of lipid-rich plaque, °	242.22 ± 86.75	221.25 ± 92.94	259.00 ± 79.86	0.199
Calcification	48(24.1%)	28(28.3%)	20(20.0%)	0.172
Angle, °	171.08 ± 84.71	188.64 ± 94.88	145.33 ± 61.28	0.129
Cholesterol crystal	120(60.3%)	65(65.7%)	55(55.0%)	0.124
Macrophage	16(8.0%)	7(7.1%)	9(9.0%)	0.617
Micro-vessel	48(24.1%)	22(22.2%)	26(26.0%)	0.533
Intracoronary thrombus	55(27.6%)	24(24.2%)	31(31.0%)	0.287
Slow/no flow	31(15.6%)	12(12.1%)	19(19.0%)	0.181
Quantitative of target vessel				
MLA, mm^2^	2.04 ± 1.08	2.12 ± 1.02	1.97 ± 1.15	0.408
MLD, mm	1.55 ± 0.40	1.59 ± 0.39	1.51 ± 0.41	0.241
Proximal reference vessel area, mm^2^	8.49 ± 3.19	8.70 ± 3.35	8.28 ± 3.02	0.418
Proximal reference vessel diameter, mm	3.30 ± 1.05	3.26 ± 0.62	3.33 ± 1.34	0.671
Distal reference vessel area, mm^2^	6.07 ± 2.38	6.44 ± 2.02	5.07 ± 2.66	0.055
Distal reference vessel diameter, mm	2.77 ± 0.71	2.83 ± 0.44	2.72 ± 0.91	0.379
Post-stent MLA, mm^2^	5.90 ± 2.41	5.74 ± 2.25	6.05 ± 2.56	0.437
Post-stent MLD, mm	2.73 ± 0.81	2.76 ± 1.01	2.71 ± 0.56	0.742
Stent diameter, mm	3.23 ± 0.47	3.25 ± 0.48	3.21 ± 0.47	0.670
Stent length, mm	25.05 ± 9.02	24.08 ± 8.60	26.01 ± 9.39	0.188
Maximal dilatation pressure, atm	20.44 ± 3.63	20.38 ± 3.83	20.50 ± 3.44	0.849
TP volume	1.81 ± 1.55	1.47 ± 1.28	2.16 ± 1.71	0.001

*SD* standard deviation, *TCFA* thin-cap fibroatheroma, *FCT* fibrous cap thickness, *MLA* minimal lumen area, *MLD* minimal lumen diameter, *TP* tissue prolapse

### Relationship between TP volume and plaque morphologies

Researchers divided the patients into two groups based on TP volume: the low TP volume group (< 1.43 mm^3^, n = 99) and the high TP volume group (≥ 1.43 mm^3^, n = 100). The OCT findings of the two groups were displayed in Table 3. The lipid arc and stent diameter were significantly larger in the high TP volume group than in the low TP volume group (P = 0.032 and P = 0.026), and the fibrous cap thickness (FCT) was smaller in the high TP volume group (P = 0.043). Likewise, the percentage of TCFA was also higher in the high TP volume group (P = 0.021), indicating the underlying relationship between TP volume and plaque vulnerability. The high TP volume group was associated with a higher percentage of lipid-rich plaque, intracoronary thrombus and slow/no flow phenomenon (All P value < 0.05). Significant differences were also observed in plaque rupture between the two groups.

**Table 3:** OCT findings of patients in low TP group and high TP group

Characteristics	All (n = 199)	Low TP (< 1.43 mm^3^, n = 99)	High TP (≥ 1.43 mm^3^, n = 100)	P value
Lipid arc, °, mean ± SD	190.00 ± 88.40	174.31 ± 94.41	205.92 ± 79.40	0.032
FCT, μm	133.60 ± 82.94	146.80 ± 97.86	119.10 ± 60.36	0.043
TCFA	57(28.6%)	21(21.2%)	36(36.0%)	0.021
Lipid-rich plaque, n(%)	97(48.7%)	44(44.4%)	53(53.0%)	0.227
Fibrous plaque	54(27.1%)	20(20.2%)	34(34.0%)	0.029
Calcification	48(24.1%)	25(25.3%)	23(23.0%)	0.710
Plaque rupture	32(16.1%)	10(10.1%)	22(22.0%)	0.022
MLA, mm^2^	2.04 ± 1.08	2.13 ± 1.19	1.95 ± 0.96	0.337
MLD, mm	1.55 ± 0.40	1.58 ± 0.43	1.52 ± 0.37	0.375
Stent diameter, mm	3.23 ± 0.47	3.14 ± 0.50	3.32 ± 0.43	0.026
Stent length, mm	25.05 ± 9.02	24.59 ± 9.21	25.51 ± 8.87	0.530
Maximal dilatation pressure, atm	20.44 ± 3.63	20.17 ± 3.41	20.71 ± 3.84	0.365
Intracoronary thrombus	55(27.6%)	21(21.2%)	34(34.0%)	0.044
Slow/no flow	31(15.6%)	10(10.1%)	21(21.0%)	0.034

*FCT* fibrous cap thickness, *TCFA* thin-cap fibroatheroma, *MLA* minimal lumen area; MLD, minimal lumen diameter

### The predictive value of Apo B/A1 for TP

**Fig. 4:**
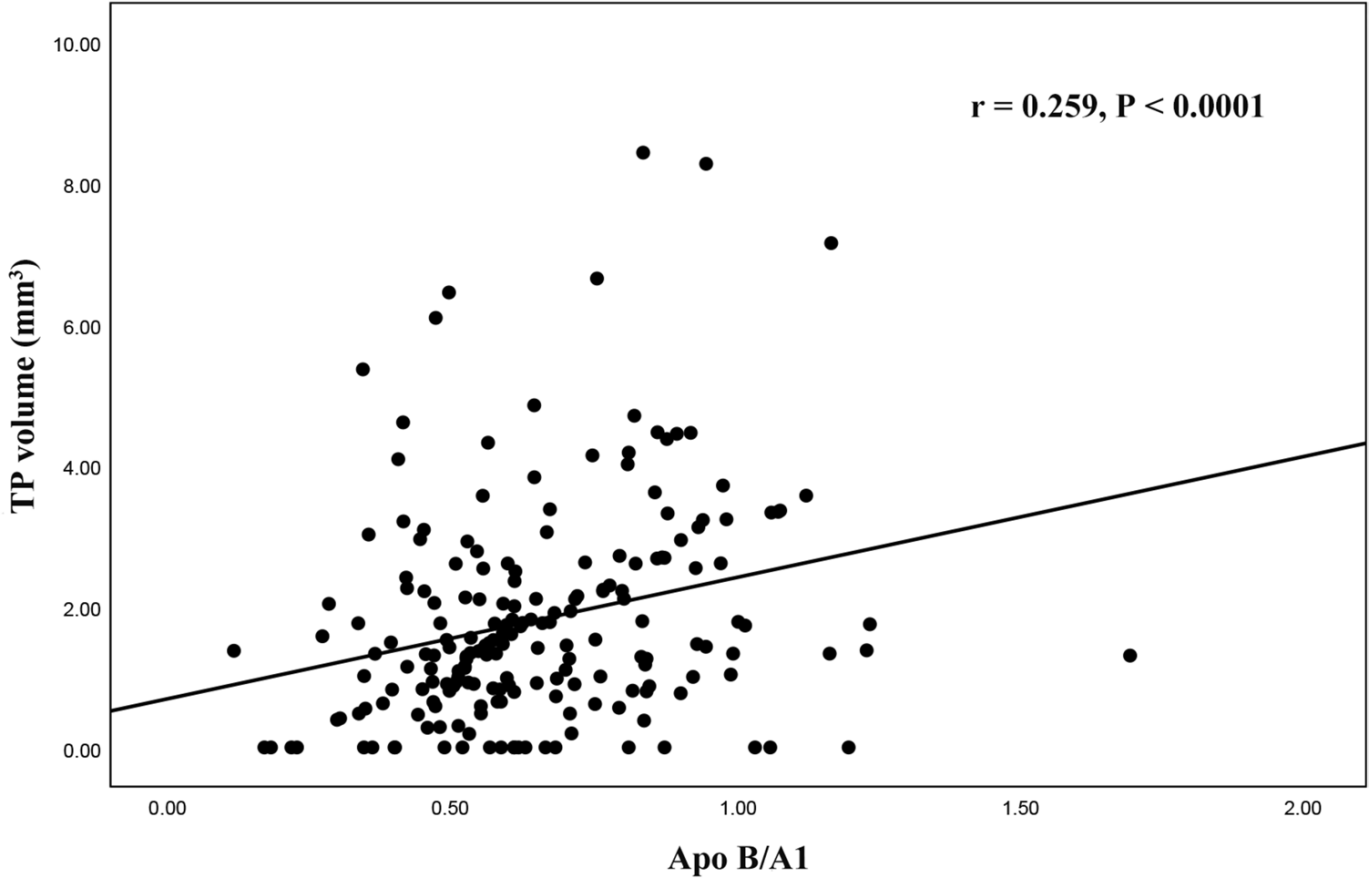
Correlation between Apo B/A1 ratio and TP volume. *Apo* apolipoprotein, *TP* tissue prolapse

**Fig. 5:**
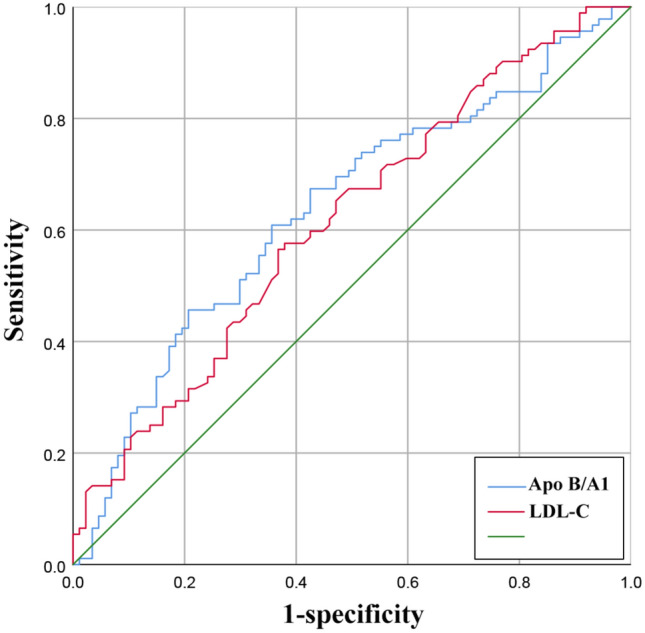
Receiver operating characteristic (ROC) curve for assessing tissue prolapse volume. *Apo* apolipoprotein, *LDL-C* low-density lipoprotein cholesterol

Multivariate logistic regression analysis demonstrated that Apo B/A1 ratio (odd ratio [OR]: 1.041, 95% confidence interval [CI] 1.007–1.076; P = 0.019), TCFA (OR: 3.199, 95%CI 1.133–9.031; P = 0.028) and intracoronary thrombus (OR: 2.866, 95%CI 1.277–6.434; P = 0.011) were significantly related to high TP volume which was shown in Table 4. Additionally, the correlation study showed that the Apo B/A1 ratio was positively related to TP volume (r = 0.259, P < 0.001) (Fig. 4). ROC curve analysis was conducted to evaluate the predictive value of the Apo B/A1 ratio for TP volume. The area under the curve (AUC) was 0.635 (95% CI 0.554–0.717, P = 0.002) for Apo B/A1 and 0.615 (95% CI 0.533–0.697, P = 0.008) for LDL-C, as shown in Fig. 5.

**Table 4:** Variables related to high TP volume (≥ 1.43mm^3^)

	Univariate analysis			Multivariate analysis		
	OR	95%CI	p value	OR	95%CI	P value
ACS	1.406	0.737–2.684	0.302			
Prior MI	0.566	0.210–1.527	0.261			
Dyslipidemia	0.710	0.346–1.454	0.349			
LDL-C	1.755	1.029–2.995	0.039	0.596	0.245–1.446	0.252
HDL-C	0.174	0.036–0.828	0.028	1.436	0.159–13.006	0.748
Apo B/A1	1.043	1.023–1.064	< 0.001	1.041	1.007–1.076	0.019
WBC count	1.177	0.996–1.391	0.055			
CRP	1.004	0.862–1.170	0.956			
LVEF	0.997	0.960–1.036	0.892			
Stent length	1.026	0.990–1.064	0.165			
Stent diameter	1.533	0.776–3.030	0.218			
Maximal dilatation pressure	1.012	0.927–1.106	0.789			
Pre-intervention MLA	0.884	0.655–1.193	0.421			
Plaque rupture	1.818	0.918–3.601	0.086			
Plaque erosion	2.739	1.361–5.510	0.005	2.013	0.917–4.419	0.081
TCFA	6.818	2.758–16.854	< 0.001	3.647	1.315–10.114	0.013
Intracoronary thrombus	2.267	1.150–4.468	0.018	2.866	1.277–6.434	0.011

*ACS* acute coronary syndrome, *TP* tissue prolapse, *MI* myocardial infarction, *LDL-C* low-density lipoprotein cholesterol, *HDL-C* high-density lipoprotein cholesterol, *WBC* white blood cell, *Apo* apolipoprotein, *CRP* C-reaction protein, *LVEF* left ventricular ejection fraction, *MLA* minimal lumen area, *TCFA* thin-cap fibroatheroma

## Discussion

This present study aimed to examine the underlying relationship among the Apo B/A1 ratio, plaque vulnerability, and TP volume in patients with atherosclerotic coronary disease. The results revealed that individuals with higher Apo B/A1 ratios had a greater incidence of plaque erosion and plaque rupture, indicating that the Apo B/A1 ratio is associated with plaque vulnerability. Additionally, quantitative analysis demonstrated a positive relationship between TP volume after PCI and Apo B/A1 ratio. Furthermore, multivariate analysis revealed that both Apo B/A1 ratio and TCFA were independently associated with TP volume. The Apo B/A1 ratio exhibited predictive value for TP volume, as evidenced by the receiver operator characteristic curve analysis. Therefore, there was underlying relationship between larger TP volume, high Apo B/A1 ratio and plaque instability.

A previous study has demonstrated that TP is an independent factor for the no-flow phenomenon after PCI in patients with acute myocardial infarction (AMI) and plaque rupture [13]. The creatine kinase-myocardial band (CK-MB) levels were greater in patients with TP lesions after stent implanting than in those without TP lesions [7, 9, 12]. Another OCT study examined the relationship between TP and neointimal proliferative restenosis and found that TP volume could predict the degree of neointimal hyperplasia [14].

Fibrous cap thickness is thought to be a resistive factor against tissue prolapse resulting from high mechanical stress during stent expansion that can lead to plaque rupture and tissue prolapse [15]. An OCT study confirmed that the plaques in patients with acute coronary syndrome exhibited more vulnerable features than those with non-acute coronary syndromes, such as wider lipid arc, longer lipid length, thinner fibrous cap, and more TCFA [16]. This study found that the fibrous cap thickness was smaller in the high TP volume group and the percentage of plaque rupture was also larger in the high TP volume group, which was consistent with the above theory and study.

While OCT has a higher resolution than IVUS [17] and can accurately identify plaque components and microstructure, it is an invasive procedure with inevitable limitations such as high cost, and potential complication risks [5]. Therefore, effective biomarkers that can predict TP need to be explored. This study quantitatively analyzed TP volume and plaque morphologies using OCT and performed a logistic analysis to determine independent factors of the severity of tissue prolapse. The results showed that TCFA and Apo B/A1 ratio were associated with the severity of tissue prolapse after excluding some clinical and imaging factors. Recent studies showed that the apo B/A1 ratio may reflect the cholesterol balance and the risk of major adverse cardiovascular events. An observational study found that the Apo B/A1 ratio is an independent predictor for complicated lesions and future myocardial infarction in patients with diabetes and acute coronary syndrome, which was consistent with our findings [18]. This study also revealed that HbA1c in the high Apo B/A1 ratio group was significantly higher than that in the low Apo B/A1 ratio group. There was a significant association between the Apo B/A1 ratio and the severity of coronary artery stenosis detected by multidetector computed tomography or coronary angiography [19–21]. The Apo B/A1 ratio was associated with diacron-reactive oxygen metabolites, which can reflect oxidative stress, endothelial dysfunction, and inflammation and high C-reaction protein level with high Apo B/A1 ratio was related to a high risk of ASCVD [22, 23]. Therefore, the Apo B/A1 ratio plays a crucial role in the genesis and development of ASCVD, and the ratio demonstrated good predictive value for the prognosis of patients with ASCVD [24].

Correlation study showed that the Apo B/A1 ratio was positively related to TP volume, and the results revealed that a higher Apo B/A1 ratio may reflect a bigger TP volume. Furthermore, the ROC curve of Apo B/A1 demonstrated predictive value for bigger TP volume and the AUC of the ROC curve of Apo B/A1 was even higher than that of LDL-C which can help to explain the high residual cardiovascular risk [2]. In patients with ACS, especially ST segment elevation myocardial infarction (STEMI), more patients had a large amount of thrombus [25]. However, even using OCT, it can sometimes be difficult to determine the difference between plaque prolapse and thrombus prolapse. To lessen the influence of thrombus prolapse, some AMI patients who potentially had a greater amount of atherosclerotic plaques and intracoronary thrombus were excluded in this study. This may explain the discrepancy that ACS patients don’t have larger tissue prolapse volume in our study.

There are inevitably several limitations of this study that should be acknowledged. First, this was a single-center, retrospective study with small sample size. Second, patients with poor OCT imaging quality and without post-OCT examination were excluded, which may result in selection bias. Third, data regarding short and long-term outcomes are lacking in this study. Finally, follow-up OCT and laboratory examination were not performed to explore the effects of TP on myocardial damage and in-stent restenosis. Thus, future studies will enroll more samples, follow-up OCT examinations and data regarding outcomes also need to be collected.

## Conclusion

This study demonstrated that TP volume was larger in the high Apo B/A1 ratio group, and the presence of TCFA was significantly related to high TP volume. The Apo B/A1 ratio is an independent predictor for TP volume on OCT which was related to plaque vulnerability.

## Data Availability

The data used and analysed during the current study are available from the corresponding author on reasonable request.

## Abbreviations

ASCVD: Atherosclerotic cardiovascular disease;
Apo: Apolipoprotein
TP: Tissue prolapse
OCT: Optical coherence tomography
PCI: Percutaneous coronary intervention
ROC: Receiver operator characteristic
TCFA: Thin-cap fibroatheroma
OR: Odds ratio; CI: confidence interval
AUC: Area under the curve
LDL-C: Low density lipoprotein cholesterol
IVUS: Intravascular ultrasound
HDL-C: High-density lipoprotein cholesterol
TC: Total cholesterol
TG: Triglycerides
SD: Standard deviation
LVEF: Left ventricular ejection fraction
FCT: Fibrous cap thickness
AMI: Acute myocardial infarction
CK-MB: Creatine kinase-myocardial band
CABG: Coronary artery bypass grifting
WBC: White blood cell
Hb: Hemoglobin
HbA1c: Glycosylated hemoglobin
LAD: Left anterior descending artery
LCX: Left circumflex artery
RCA: Right coronary artery
MLA: Minimal lumen area
MLD: Minimal lumen diameter
